# Antifungal Activity of Quinofumelin against *Fusarium graminearum* and Its Inhibitory Effect on DON Biosynthesis

**DOI:** 10.3390/toxins13050348

**Published:** 2021-05-12

**Authors:** Qian Xiu, Lianyu Bi, Haorong Xu, Tao Li, Zehua Zhou, Zhongke Li, Jianxin Wang, Yabing Duan, Mingguo Zhou

**Affiliations:** 1 Department of Pesticide Science, College of Plant Protection, Nanjing Agricultural University, Nanjing 210095, China; 2018102140@njau.edu.cn (Q.X.); 2019102114@njau.edu.cn (L.B.); imvikii@163.com (H.X.); litaolitao1990@126.com (T.L.); m15895982813@163.com (Z.Z.); 13653760372@163.com (Z.L.); jlz489@163.com (J.W.); 2 The Key Laboratory of Plant Immunity, College of Plant Protection, Nanjing Agricultural University, Nanjing 210095, China

**Keywords:** control efficacy, DON biosynthesis, *Fusarium graminearum*, Fusarium head blight, quinofumelin

## Abstract

*Fusarium graminearum,* causal agent of Fusarium head blight (FHB), causes a huge economic loss. No information is available on the activity of quinofumelin, a novel quinoline fungicide, against *F. graminearum* or other phytopathogens. In this study, we used mycelial growth and spore germination inhibition methods to determine the inhibitory effect of quinofumelin against *F. graminearum* in vitro. The results indicated that quinofumelin excellently inhibited mycelial growth and spore germination of *F. graminearum*, with the average EC_50_ values of 0.019 ± 0.007 μg/mL and 0.087 ± 0.024 μg/mL, respectively. In addition, we found that quinofumelin could significantly decrease deoxynivalenol (DON) production and inhibit the expression of DON-related gene *TRI5* in *F. graminearum*. Furthermore, we found that quinofumelin could disrupt the formation of Fusarium toxisome, a structure for producing DON. Western blot analysis demonstrated that the translation level of TRI1, a marker gene for Fusarium toxisome, was suppressed by quinofumelin. The protective and curative assays indicated that quinofumelin had an excellent control efficiency against *F. graminearum* on wheat coleoptiles. Taken together, quinofumelin exhibits not only an excellent antifungal activity on mycelial growth and spore germination, but also could inhibit DON biosynthesis in *F. graminearum*. The findings provide a novel candidate for controlling FHB caused by *F. graminearum*.

## 1. Introduction

Fusarium head blight (FHB), primarily caused by *Fusarium graminearum* species complex (FGSC), is one of the major diseases in wheat in the wheat growing regions of the world [1,2]. FHB pathogens can invade wheat at different stages of growth, causing seedling rot, stem rot or wheat ear rot. In the field, FHB pathogens mainly invade the panicle, causing rot of the spikelet or the whole wheatear, resulting in the shrinkage of kernels or head blight. In China, severe FHB epidemics have occurred more frequently since 2010. More than 5.4 Mha, which accounts for about 23% of the total wheat production area of China, were affected by the disease each year between 2000 and 2018 [3]. In America, the wheat production caused by FHB was reduced up to 501 million bushels in the early 1990s [2]. Since 2000, FHB has been more frequent and severe in the Great Plains, a region which had most of the hard winter wheat. FHB epidemics resulted in the loss of up to 70% in Argentina in 2012 and resulted in more than 80% of infected spikes in some production fields in Georgia between 2014 and 2016 [4,5]. In addition, FHB pathogens can produce a variety of mycotoxins in the grains, including deoxynivalenol (DON), zearalenone (ZEN), and nivalenol (NIV). The mycotoxins secreted by *F. graminearum* have some serious toxic effects on animals and human beings, such as feed refusal, inhibition of protein synthesis, cardiotoxicity, teratogenicity, immunotoxicity and organ toxicity [6,7,8]. Due to the lack disease-resistant varieties and successful cases of biological control that can be used in the field, the control of FHB is still dominated by chemical control [9].

Over the decades, carbendazim has played an important role in the control of FHB in China [10]. However, due to its single action site and its long-term use, carbendazim-resistant strains have appeared and increased continuously in the field [11]. Unfortunately, carbendazim resistance not only caused control failure of benzimidazoles in FHB [12,13], but also aggravated the contamination of mycotoxins in wheat grains [14]. Phenamacril has a special mode of action and showed a great inhibitory activity against *F. graminearum*. Moreover, there is no cross-resistance between phenamacril and benzimidazoles, so it can replace benzimidazoles such as carbendazim to control FHB caused by carbendazim-resistant *F. graminearum* [15]. However, it is easy to develop phenamacril resistance in *F. graminearum* by ultraviolet irradiation or chemical taming in vitro and most resistant strains were medium resistant (MR) or of a high resistant level (HR). Therefore, large-scale application of phenamacril will have a high risk of resistance in the field [16]. Sterol biosynthesis inhibitor (SBI) fungicides, including tebuconazole, epoxiconazole and metconazole, exhibited an excellent antifungal activity against FHB pathogens in vitro and an excellent control efficacy on FHB in the field [12,17]. However, due to extensive use, tebuconazole-resistant strains have appeared in some provinces of China [18]. Therefore, it is necessary to find alternative fungicides with new modes of action to control FHB in China.

**Figure 1:**
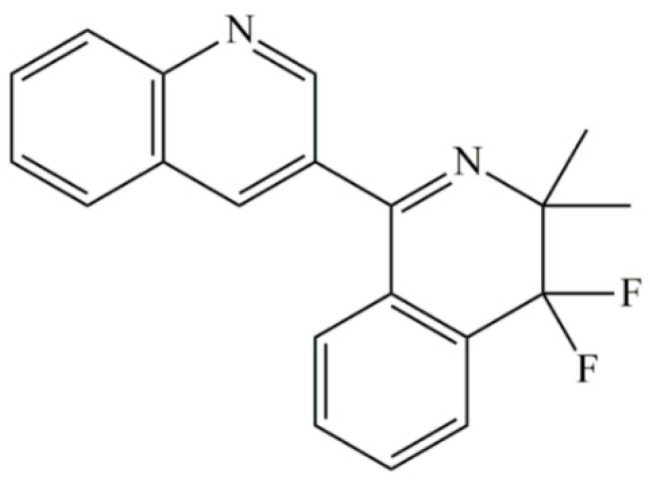
The chemical structure of quinofumelin.

Quinofumelin (test code: ARK-3010, Cas No. 861647-84-9) is a novel quinoline fungicide developed by Mitsui Chemicals Co. Ltd., Japan (Figure 1). Quinoline compounds and their derivatives have been widely used in medicine and pesticides due to their high bioactivity [19,20]. Quinolines are classified as FRAC group 13 based on their biological and physiological characteristics [21]. However, the mode of action of quinolines against plant pathogens is unclear. In addition, limited data on antifungal activity of quinofumelin against plant pathogens are available. Recently, quinofumelin has been found to exhibit an excellent antifungal activity against *Sclerotinia sclerotiorum* [22]. To date, the bioactivity of quinofumelin against *F. graminearum* is not reported. In this study, baseline sensitivity of *F. graminearum* populations from different geographic regions in China to quinofumelin was established based on mycelial growth inhibition and spore germination methods; the effects of quinofumelin on DON biosynthesis and toxisome formation were determined and control efficacy in planta of quinofumelin against *F. graminearum* was evaluated. These findings not only provide important references for quinofumelin being applied in FHB control, but also provide invaluable insights for uncovering an action mode of quinofumelin against plant pathogens.

## 2. Results

### 2.1. The Sensitivity of F. graminearum to Quinofumelin

**Figure 2:**
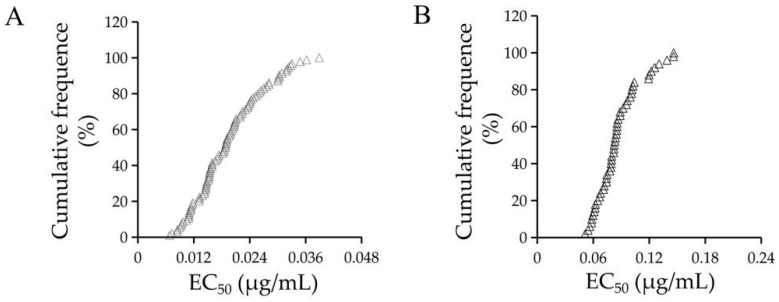
Sensitivity distribution of *F. graminearum* isolates to quinofumelin based on a mycelial growth inhibition method (**A**) and a spore germination inhibition method (**B**). Isolates are ranked according to increasing EC_50_ values (cumulative).

In this study, the sensitivity tests of *F. graminearum* to quinofumelin were performed based on mycelial growth and spore germination inhibition methods. For mycelial growth, a total of 100 *F. graminearum* isolates from the diseased wheat ears were used to test the sensitivity to quinofumelin. The EC_50_ values of quinofumelin ranged from 0.007 to 0.039 μg/mL, with an average EC_50_ value of 0.019 ± 0.007 μg/mL (Figure 2A). For spore germination, 50 *F. graminearum* isolates were selected and tested. The EC_50_ values of quinofumelin ranged from 0.051 to 0.205 μg/mL, with an average EC_50_ value of 0.087 ± 0.024 μg/mL (Figure 2B). These results suggested that quinofumelin exhibited a high antifungal activity on either mycelial growth or spore germination of *F. graminearum*.

### 2.2. Quinofumelin Reduced DON Biosynthesis and TRI5 Gene Expression in F. graminearum

**Figure 3:**
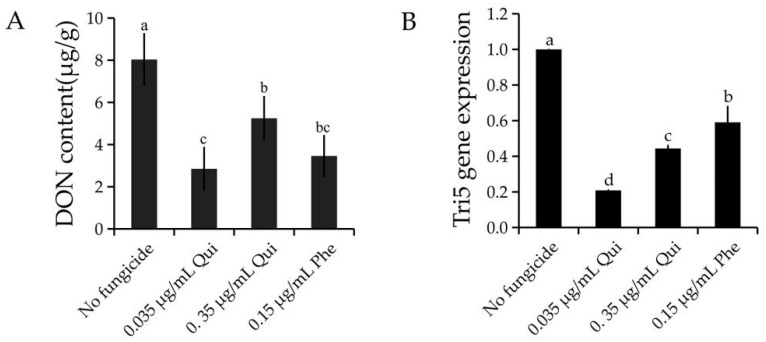
DON production (**A**) and *TRI5* gene expression (**B**) in *F. graminearum* affected by quinofumelin. Qui: quinofumelin. Phe: Phenamacril. The final concentrations are 0.035, 0.35 μg/mL of quinofumelin and 0.15 μg/mL of phenamacril, respectively. Values are the mean and standard errors of three replicates. Letters above the column showed the difference of different treatments (*p* < 0.05, ANOVA, LSD).

The EC_50_ and 10 × EC_50_ values of the wild type isolate PH-1 were 0.035 and 0.35 μg/mL, respectively. To assess whether quinofumelin has an inhibitory effect on DON production in *F. graminearum*, extracellular DON in TBI medium was assessed with a DON toxins kit according to the manufacturer’s protocol. The results showed that both 0.035 and 0.35 μg/mL of quinofumelin significantly reduced DON production in comparison to the untreated controls (Figure 3A). The *TRI5* gene is pivotal in the DON biosynthesis pathway in *F. graminearum* [23]. To confirm the effect of quinofumelin on the expression of the *TRI5* gene, we measured the expression of *TRI5* by quantitative real-time PCR (qRT-PCR). Compared to the untreated controls, the expression of the *TRI5* gene was significantly down-regulated by quinofumelin (Figure 3B). Moreover, the positive control fungicide phenamacril could reduce DON production and down-regulate *TRI5* gene expression in *F. graminearum*. The above-described results strongly indicated that quinofumelin significantly reduced DON biosynthesis in *F. graminearum.*

### 2.3. Quinofumelin Affects the Formation of Toxisomes and the Expression of TRI1 in the Translational Level in F. graminearum

**Figure 4:**
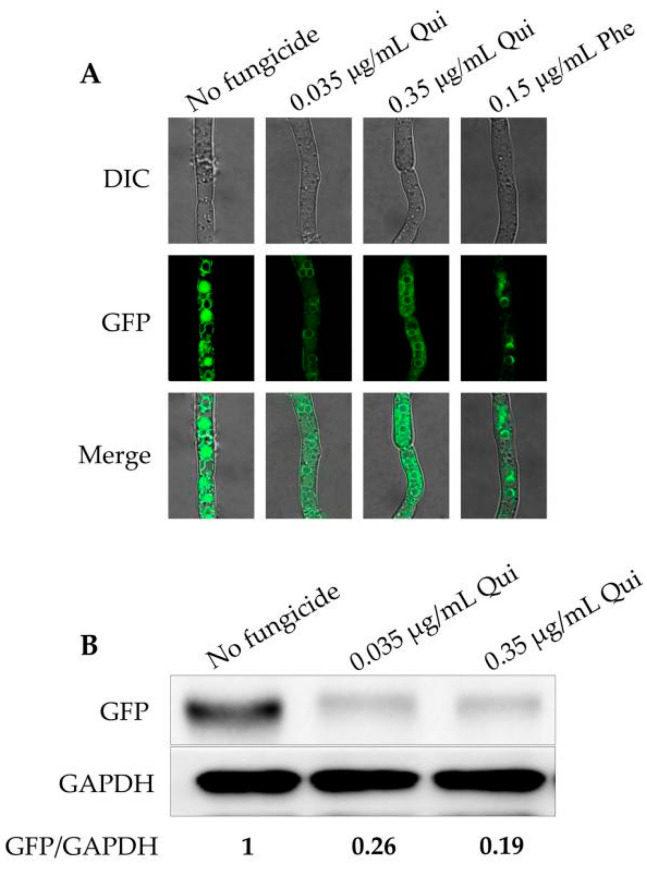
Formation of toxisomes and the translation level of TRI1 as affected by quinofumelin. Qui: quinofumelin. Phe: Phenamacril. (**A**) Toxisomes in the mycelia of the strain FgTri1-GFP after treatment with 0.035 μg/mL and 0.35 μg/mL of quinofumelin or 0.15 μg/mL phenamacril for 24 h in TBI medium. (**B**) Translation level of TRI1 after treatment with quinofumelin for 2 days. GAPDH was used as a reference.

Previous studies have demonstrated that calonectrin oxygenase (Tri1) is a key enzyme of the TRI pathway to regulate downstream reactions and the *TRI1* gene localized in toxisomes, a special spherical structure formed in *F. graminearum* [24,25,26]. To measure the impact of quinofumelin on the formation of toxisomes in *F. graminearum*, TRI1 was labeled with a GFP tag and visualized with a laser confocal microscope (Leica TCS SP5). The results showed that the fluorescence intensity of TRI1 with quinofumelin treatments were weaker than untreated controls (Figure 4A). To further confirm the effect of quinofumelin on the translation level of the *TRI1* gene, the GFP-tagged isolate was grown in TBI medium for 3 days and the translation level of TRI1 was analyzed by Western blot. The results showed that both 0.035 μg/mL and 0.35 μg/mL of quinofumelin reduced the expression of TRI1 in comparison to the untreated controls (Figure 4B). All the above-described results indicated that quinofumelin disrupts the formation of toxisomes and decrease the expression of TRI1 in *F. graminearum*.

### 2.4. Protective and Curative Activity of Quinofumelin against FHB

**Figure 5:**
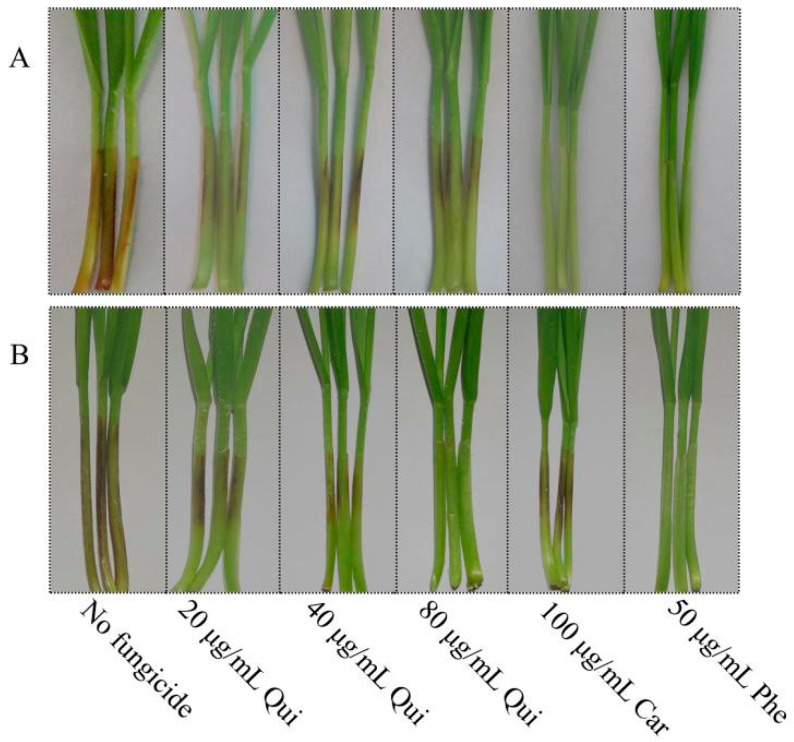
Protective (**A**) and curative activities (**B**) of quinofumelin against *F. graminearum* on wheat coleoptiles. Each coleoptile was injected with 2.5 μL of conidia (1 × 10^6^ mL^−1^) and was maintained in the illumination incubator for 10 days. Qui: quinofumelin. Car: carbendazim. Phe: phenamacril.

Protective and curative activity of quinofumelin against *F. graminearum* on wheat coleoptiles were determined. For protective activity, control efficacy of quinofumelin increased as the increase of use dosage, but did not differ between 40 μg/mL and 80 μg/mL quinofumelin. Control efficacy for 80 μg/mL quinofumelin reached 81.05%, lower than that for 100 μg/mL carbendazim and 50 μg/mL phenamacril (Table 1, Figure 5). For curative activity, control efficacy of quinofumelin increased as the increase of use dosage. Control efficacy for 80 μg/mL quinofumelin reached 92.93%, equivalent to that for 50 μg/mL phenamacril (Table 1, Figure 5). However, carbendazim had a poor control efficacy for curative activity as compared to protective activity (Table 1, Figure 5). All the results suggested that quinofumelin had good protective and curative activities against *F. graminearum* in wheat coleoptiles.

## 3. Discussion

*F. graminearum* is a destructive pathogen on various cereals, and the epidemic of FHB caused by *F. graminearum* is a disaster for grains. Besides the yield loss, FHB also reduces grain quality by producing mycotoxins. Deoxynivalenol (DON) is one of the principal mycotoxins produced by *F. graminearum*, which is unsafe for human consumption, animal feed and malting produce and poses a serious threat to food safety [7]. As most wheat cultivars are susceptible to *Fusarium* species, the primary method to manage FHB is fungicide application. Benzimidazole fungicides, particularly carbendazim, have been widely used for the control of FHB in China for several decades. Unfortunately, resistance to carbendazim has already been widespread in China because of its extensive application, leading to control failure of FHB. Moreover, previous studies have reported that carbendazim resistance could accelerate DON biosynthesis in *F. graminearum* [27], aggravating the risk of food safety. Phenamacril is a *Fusarium*-specific fungicide and has been reported to inhibit DON biosynthesis in *F. graminearum* [28]. However, phenamacril resistance was relatively easy to develop in *Fusarium* species [29]. Thus, it is urgent to find novel fungicides to control FHB and decrease DON contamination caused by *F. graminearum*.

Quinoline compounds are ubiquitous nitrogen-containing aromatic heterocycles that have been reported to be applied in industrial and medicinal fields [30]. Previous studies have reported that some quinoline derivatives exhibited good biological activities. For example, quinoxyfen is an effective control agent for powdery mildew [19], and tebufloquin showed high activity on rice blast [19]. Quinofumelin, a special and novel quinoline fungicide, has been reported to exhibit an excellent antifungal activity against *S. sclerotiorum* [22]. We found that quinofumelin showed great inhibitory effects on mycelial growth and spore germination in *F. graminearum*. In this study, the sensitivity of 100 *F. graminearum* isolates and 50 *F. graminearum* isolates to quinofumelin was determined with mycelia growth and spore germination inhibition methods, respectively. The average EC_50_ value for mycelial growth of 100 *F. graminearum* isolates was 0.019 ± 0.007 μg/mL. To our knowledge, the mean EC_50_ value for quinofumelin-inhibiting mycelial growth of *F. graminearum* was lower than phenamacril, tebuconazole, carbendazim, metconazole, and epoxiconazole [12,17,18,31,32]. Previous studies reported that Quinone outside inhibitors (QoIs) and Succinate dehydrogenase inhibitors (SDHIs) had good effects in the control of FHB. For example, azoxystrobin and fluopyram can effectively inhibit spore germination, with the EC_50_ value from 0.274 to 1.240 μg/mL, 0.39 to 0.74 μg/mL, respectively [23,33]. Compared to azoxystrobin and fluopyram, quinofumelin had a better inhibition activity on spore germination of *F. graminearum* in vitro. In the field, the ascospores released from matured perithecia are the primary infectious source and the spores from the infected wheat ears continually complete the infection, causing disease burst. Quinofumelin, as a new quinoline compound, showed an excellent inhibitory activity on either mycelial growth or spore germination. Additionally, our findings also indicated that quinofumelin exhibited great protective and curative effects against *F. graminearum* in wheat coleoptiles. When the concentration of quinofumelin increased to 80 μg/mL, the control efficacies for protective and curative effects reached 81.05% and 92.93%, respectively. Compared with carbendazim, quinofumelin has a better curative activity. Although phenamacril exhibits a specific protective and curative activity against FHB, it has a high resistance risk in FGSC [31].

DON is a pernicious secondary metabolite produced by *F. graminearum* that can decrease grain quality and is harmful to the health of humans and animals. Thus, in addition to high antifungal activity, inhibitory effect of DON production is also an essential indicator to evaluate whether one fungicide can be applied in the field. In the current study, we found that quinofumelin strongly reduced DON production. At present, the biosynthetic pathway of DON has been extensively studied, and nearly all the DON biosynthesis-involved genes have been identified [24]. The *TRI5* gene is pivotal in DON biosynthesis in *F. graminearum* [34]. We found that the expression level of the *TRI5* gene was significantly reduced by quinofumelin. Furthermore, *Tri1*, the key DON biosynthetic enzyme, is mainly localized to the toxisomes derived from endoplasmic reticulum under toxin inducing conditions [28]. In this study, quinofumelin disrupted the formation of toxisomes and decreased *TRI1* expression. The results revealed that quinofumelin not only exhibited an inhibitory effect on spore germination and mycelial growth of *F. graminearum*, but also decreased DON biosynthesis. To our knowledge, this is the first report that quinofumelin inhibits fungal growth and DON biosynthesis in *F. graminearum.*

In conclusion, quinofumelin exhibited a great inhibitory effect on the mycelial growth and spore germination in *F. graminearum*. Moreover, quinofumelin can also decrease DON biosynthesis in vitro. All the results will provide valuable information for a wheat protection program against FHB. However, its resistance risk is still unknown, and should be further studied.

## 4. Materials and Methods

### 4.1. Fungal Growth and Culture Conditions

*F. graminearum* isolates were isolated from the diseased wheat ears from Jiangsu Province, China. All of the isolates were maintained on PDA slants and stored at 4 °C.

Potato dextrose agar (PDA) medium was made from 200 g of potato, 20 g of glucose, 16 g of agar powder per liter of distilled water. Water Agar (WA) medium consisted of 15 g agar powder per liter of distilled water. Mung bean broth (MBB) medium consisted of 30 g green bean per liter of distilled water. Trichothecene biosynthesis induction (TBI) medium was made from 30 g sucrose, 2 g NaNO_3_, 1 g KH_2_PO_4_, 0.5 g MgSO_4_·7H_2_O, 0.5 g KCl, 10 mg FeSO_4_·7H_2_O, 0.3 g phytagar, 0.871 g L-Arginine, 10 mg ZnSO_4_·7H_2_O, 0.5 mg CuSO_4_·5H_2_O, 0.1 mg MnSO_4_·H_2_O, 10 mg citric acid, 0.1 mg H_3_BO_3_, 0.1mg NaMoCl_4_·2H_2_O, pH = 6.5 per liter of distilled water [33].

### 4.2. Fungicides

Chemical-grade quinofumelin (Mitsui Agricultural Chemical Company of Japan) was prepared with dimethyl sulfoxide in 1 × 10^4^ mg/L, 1 × 10^3^ mg/L, 1 × 10^2^ mg/L, Technical-grade phenamacril (Jiangsu Branch of National Pesticide Research and Development South Center of China) was prepared with dimethyl sulfoxide in 1 × 10^3^ mg/L, 1 × 10^2^ mg/L and carbendazim (Shenyang Academy of Chemistry and Industry, China) was dissolved in dimethyl sulfoxide at 1 × 10^3^ mg/L. All the reagents were stored at 4 °C. Chemical- and technical-grade fungicides were used for mycelial growth sensitivity, spore germination, Western blot, DON production and the virulence assay.

### 4.3. Fungicide Sensitivity Test

For mycelial growth, 100 strains of *F. graminearum* were tested. Mycelial plugs (5 mm diameter) from the edge of 3-day-old colonies of each isolate were placed on PDA plates containing 0, 0.01, 0.02, 0.04, 0.08, and 0.16 μg/mL of quinofumelin. After the plates were cultured in an incubator at 25 °C for 3 days, the diameters of the colonies were measured and the EC_50_ values were calculated based on a linear regression of colony diameters on log-transformed fungicide concentration. Three replicates were performed for each concentration. For spore germination, 50 isolates were tested. Spores were cultured in MBB medium at 25 °C for 3 days and harvested with a lens paper of 3 layers. Then the spores were rinsed with sterile water and were adjusted to the concentration of 1 × 10^6^ spores/mL. Petri dishes of 9 cm were filled with 15 mL of WA medium containing 0, 0.01875, 0.0375, 0.075, 0.15, and 0.3 μg/mL of quinofumelin. Aliquots of 30 μL of the spores were dropped on the quinofumelin-amended WA plates. After incubation in the dark at 25 °C for 7 h, the germinated spore was determined if the germ tube was at least half the length of the spore and 100 spores were examined for each petri dish. Three replicates for each concentration were used. The EC_50_ values were calculated based on linear regression of spore germination rates on log-transformed fungicide concentration.

### 4.4. DON Biosynthesis In Vitro

DON is an important virulence factor of *F. graminearum* [35]. The wild-type isolate PH-1 was chosen to explore the impact of quinofumelin on DON production in vitro. Spores (1 × 10^4^) were added into 20 mL TBI according to the previous study [14]. After being incubated in the dark at 28 °C for 24 h, quinofumelin was added to the TBI medium to the final concentrations of 0.035 μg/mL and 0.35 μg/mL. Phenamacril at 0.15 μg/mL of a final concentration (EC_50_ value) was used as the positive control. After being incubated for an additional 6 days, the mycelia were harvested, dried, and weighed and the culture fluid were collected to determine DON production with DON Determination ELISA Kit (Weisai, Zhenjiang, China). DON production (μg/g) was evaluated as a ratio of DON content to the dry weight of mycelia. Each treatment had three replicates and the experiments were performed three times.

For *Tri5* gene expression, the spores of the isolate PH-1 were added to the TBI medium (5 × 10^4^ spores in 100 mL TBI). After being incubated in the dark at 28 °C for 24 h, quinofumelin and phenamacril were added to the medium at the described-above concentrations. After incubation for an additional 2 days, total RNA was extracted with Total RNA Extraction Kit (Tiangen, Beijing, China). Reverse transcription PCR was performed with the HiScript Ⅱ qRT SuperMix for qPCR (+gDNA wiper) (Vazyme, Nanjing, China) as described in Zhou et al. [36] Primers used for qRT-PCR were listed in Table 2. The expression of the glyceraldehyde-3-phosphate dehydrogenase (*GAPDH*) gene of *F. graminearum* was used as the reference gene. The relative expression level of each gene was calculated with the 2^−ΔΔCt^ method. The experiments were performed three times with three replicates for each treatment.

### 4.5. Formation of Toxisomes and Western Blotting Assays

The strain FgTri1-GFP labeled with Tri1-GFP in the ΔTri1 background was constructed as previous described [28,33]. The strain was cultured in TBI medium in the dark at 28 °C for 24 h, quinofumelin and phenamacril were added to the medium as described above and incubated for a further 24 h. All samples were placed on glass slides and sealed with cover slides. The localization of the tagged protein TRI1 and the formation of toxisomes was observed with Leica TCS SP5 laser confocal microscope (Wetzlar, Hessen, Germany). For observation of the GFP-tagged TRI1, the excitation wavelength of 488 nm was used.

For Western blot assays, the strain FgTri1-GFP was incubated in TBI medium in the dark at 28 °C for 24 h, and then quinofumelin was added to the TBI medium as described above. The medium was incubated for an additional 48 h and the mycelia were harvested and washed with sterile water three times. The method of protein extraction was conducted according to a previous study [14]. The translation level of the strain FgTri1-GFP as affected by quinofumelin was further confirmed with Western blot. The extracted protein was separated in 10% SDS-PAGE gels and transferred to Immobilon-P transfer membrane (Millipore, Billerica, MA, USA). The monoclonal anti-GFP antibody (Cat. No. 300943, ZENBIO, Chengdu, China) was used at a 1:1000 dilution. For detection of the reference protein GAPDH, the monoclonal anti-GAPDH antibody (Cat. No. 60004, Proteintech) was used. Incubation with a secondary antibody and chemiluminescent detection were performed as described previously [37]. The intensity of immunoblot bands were quantified with the ImageJ (1.8.0. Bethesda, MA, USA).

### 4.6. Protective and Curative Activity of Quinofumelin on Wheat Coleoptiles

Wheat seeds of Huaimai 33 were vernalized at an illumination incubator for 24 h. The germinated seeds were incubated on 2 layers of wetted filter paper for 30 seeds per dish and incubated in a chamber (25 °C, 75% humidity) with 12 h of light and 12 h of dark. At the same time, PH-1 was incubated in MBB medium at 25 °C for 3 days to produce spores, the spores were harvested by filtering with three layers of filter papers and were centrifuged at 8000 rpm for 10 min. The harvested spores were subsequently re-suspended in sterile distilled water and diluted to a concentration of 1 × 10^6^ spores/mL. An aliquot of 2.5 mL spore suspension was injected at the top of the wheat coleoptiles. After incubation for 7 days, lesion length was examined and control efficacy was calculated as described previously [38]. The stock solution was diluted with water containing 0.1% tween 20 to 20 mg/L, 40 mg/L, 80 mg/L of quinofumelin, 100 mg/L of carbendazim and 50 mg/L of phenamacril. For the protective activity assay, at 24 h before inoculation, the fungicides were sprayed on the coleoptiles until liquid flowed on the surface. For the curative activity assay, 24 h after inoculation, fungicides were sprayed on the coleoptiles until liquid flowed on the surface [13]. The no-fungicide treatments were the same as the controls. The plants were kept in the chamber (25 °C 75% humidity). After incubation for an additional 7 days, the length of lesions on the coleoptiles in each inoculated wheat seedling was measured. The experiments were performed three times with three replicated dishes (30 seedlings per dish) of each concentration.

### 4.7. Data Analysis

Data in the study were subjected to analysis of variance (ANOVA) with the SPSS 14.0 software (SPSS Inc. Chicago, IL, USA). When ANOVA was significant (*p* = 0.05), means were separated with Fisher’s protected least significant difference (PLSD).

## Figures and Tables

**Table 1:** Protective and curative activity of quinofumelin against *F. graminearum* on wheat coleoptiles.

Fungicide	Concentration (μg/mL)	Protective Activity		Curative Activity	
		Lesion Length ^a^ (cm)	Control Efficacy ^a^ (%)	Lesion Length ^a^ (cm)	Control Efficacy ^a^ (%)
Quinofumelin	20	0.93 ± 0.24 ^a^	57.69 ^d^	1.04 ± 0.22 ^b^	53.58 ^c^
	40	0.48 ± 0.18 ^b^	78.19 ^c^	0.56 ± 0.11 ^c^	75.16 ^b^
	80	0.42 ± 0.18 ^b^	81.05 ^c^	0.16 ± 0.07 ^d^	92.93 ^a^
Carbendazim	100	0.16 ± 0.09 ^c^	92.44 ^b^	1.35 ± 0.24 ^a^	40.00 ^d^
Phenamacril	50	0.05 ± 0.01 ^d^	97.58 ^a^	0.09 ± 0.02 ^d^	95.98 ^a^

^a^ Values are mean ± standard error of the replicates. Means followed by the different letters in a column are significant difference according to the least significantly difference (LSD) test at *p* = 0.05.

**Table 2:** Primers used in the study.

Primer	Sequence (5’-3’)	Use
FGSG_03537-qF	GGCTTCCCTCCAAACAAT	RT-qPCR for the expression of *Tri5*
FGSG_03537-qR	TGGGAAAGTGCTCGTTGA	
GAPDH-qF	CTTACTGCCTCCACCAACTG	RT-qPCR for the internal control
GAPDH-qR	TGACGTTGGAAGGAGCGAAG	

## Data Availability

The original data are available upon request from the authors.
